# Vector-Borne Disease Control and Management in Irrigation Areas: A Neglected Critical Phenomenon in Malawi

**DOI:** 10.3390/tropicalmed10090251

**Published:** 2025-09-02

**Authors:** Levi Kalitsilo, Rose Oronje, Nyanyiwe Masingi Mbeye

**Affiliations:** 1 African Institute for Development Policy, Lilongwe P.O. Box 31024, Malawi; 2 African Institute for Development Policy, Nairobi P.O. Box 14688-00800, Kenya; rose.oronje@afidep.org; 3 Department of Epidemiology and Biostatistics, School of Global and Public Health, Kamuzu University of Health Sciences, Lilongwe P.O. Box 30184, Malawi; nyanyiwembeye@gmail.com

**Keywords:** vector-borne diseases, integrated vector management, One Health, malaria, schistosomiasis, dengue

## Abstract

Vector-borne diseases (VBDs) account for more than 17% of all infectious diseases, causing over 700,000 deaths annually, particularly among the poorest populations in tropical and subtropical areas. Climate change, particularly global warming, and certain human activities, including irrigation farming, exacerbate the situation by creating conducive environments that facilitate the breeding of vectors such as mosquitoes and snails. This qualitative study aimed to understand the VBD control and management policy landscape in irrigation areas by gathering perceptions from key stakeholders in the irrigation farming sector in Malawi. Respondents indicated that there are no specific VBD control and management policies targeting irrigation areas in Malawi and that stakeholders essentially work in silos. Notwithstanding this, the Malawi government is committed to expanding irrigation areas to address food security. We, therefore, call for the integration of VBD control and management in irrigation farming, utilising the One Health approach—a promising strategy that could bring significant benefits. Further, we recommend the provision of VBD control and management resources in irrigation investments and the involvement of VBD researchers in the formulation of irrigation policies.

## 1. Introduction

Vector-borne diseases (VBDs) are human illnesses caused by parasites, bacteria, or viruses and account for more than 17% of all infectious diseases, causing more than 700,000 deaths annually [1]. They include malaria, dengue, schistosomiasis, human African trypanosomiasis (HAT), leishmaniasis, Chagas disease, yellow fever, Japanese encephalitis, and onchocerciasis [2]. The burden of VBDs is highest in tropical and subtropical areas, disproportionately affecting the poorest populations. In its strategic approach to addressing VBDs, Global Vector Control Response 2017–2030 (GVCR), WHO estimates that over 80% of the global population is at risk of contracting at least one VBD in their lifetime [3]. In Malawi, VBDs are a major public health concern, with malaria being the most prevalent and the leading cause of mortality and morbidity among children under 5, with a prevalence of 35.4% reported in 2020 [4]. Further, schistosomiasis is among the 20 causes of outpatient visits in Malawian health facilities, with 40% to 50% of the population being at risk of infection [5].

Changes in climate and anthropogenic factors largely affect VBDs epidemiology, raising more fears as global temperatures are expected to increase by 2.7 degrees Celsius by 2075 [6]. The incidence of malaria is expected to increase across 7–9 months as opposed to the historical 3–4 months; the range is also expected to expand towards the highlands [7]. Similar trends are expected for dengue fever, as the mean relative vectorial capacity for transmission is projected to increase from about 0.47 to about 0.61 if emissions do not decrease [8]. While irrigated farming has been seen as one way of ensuring food security, especially in drought-prone areas, there are indications that it creates additional vector breeding sites, thereby enhancing VBD transmission, such as malaria [9,10,11]. For example, some studies have documented increased malaria risk due to rice irrigation, mainly demonstrating its impact using entomological parameters [12,13]. Rice irrigation may also alter the transmission dynamics of malaria from seasonal to perennial [14], mainly as a result of the prolonged presence of aquatic habitats suitable for vector breeding. Vector control is, therefore, key to reducing the burden of VBDs [15], but such interventions are often riddled with implementation challenges. The vertical administration of VBD control programmes may result in duplication of efforts and, therefore, wastage of resources. The WHO recommends Integrated Vector Management (IVM) as a rational process for addressing VBD control and management [16]. It is a cross-cutting intervention that has emerged as an effective way of preventing VBDs [1]. IVM involves using a range of proven vector control methods, either in combination or alone; several methods against a single disease; or single or several methods against several diseases, and is achieved through joint multi-sectoral planning, implementation, enforcement, and validation of vector control efforts from the relevant national sectors and stakeholders [16]. An integrated, unifying approach aimed at sustaining balance and optimising the health of people, animals and ecosystems known as ‘One Health’ is required when implementing IVM to address the diverse types of interactions and often complex cycles of transmission of VBDs between humans and vectors (animals), as well as the changing social and environmental conditions [17].

In Sub-Saharan Africa, irrigation schemes have been constructed or are under construction to address increasing food demand [1]. The Shire Valley Transformation Project (SVTP) in Southern Malawi is one such project being implemented by the Ministry of Agriculture and with support from the World Bank, African Development Bank, and the Global Environment Facility (GEF). The 14-year-long project involves irrigating 43,370 hectares of land by abstracting water from the Shire River, the largest river in Malawi, and conveying it to the irrigable area in Chikwawa and Nsanje Districts through canals. Water resource management projects like this are among the factors that can impact vectors’ ecology and transmission patterns [10]. A shift in VBD activity has socioeconomic implications as it burdens healthcare systems and increases health inequity. Hence, there is a need to understand the policy landscape and perceptions of policymakers and implementers on irrigation farming and VBD control and management in Malawi.

The expansion of irrigation schemes in Malawi, particularly in the Lower Shire Valley, is likely to introduce new ecological conditions that heighten the risk of vector-borne diseases (VBDs). While irrigation is essential for improving agricultural productivity and climate resilience, it inadvertently creates breeding grounds for disease vectors such as *Anopheles* mosquitoes and freshwater snails, thereby intensifying transmission of malaria and schistosomiasis [18,19]. Recent findings from the Shire Valley Vector Control Project (Shire-Vec) underscore the urgency of integrating vector control strategies into irrigation planning to mitigate these health risks [18]. Without deliberate public health safeguards, large-scale irrigation initiatives may exacerbate the burden of VBDs, undermining both health and development goals in vulnerable communities.

The African Institute for Development Policy (AFIDEP), Liverpool School of Tropical Medicine (LSTM), the Malawi Liverpool Wellcome Trust Programme (MLW), and the Malaria Alert Centre (MAC) at the Kamuzu University of Health Sciences (KUHeS) carried out a qualitative study as part of the Shire Valley Vector Control Project to examine the impact of agricultural expansion on vector-borne diseases (VBDs) in the Shire Valley and other selected areas in Malawi. The study explored the experiences, practices, and perspectives regarding VBD control and management in irrigation schemes. This aimed to create the evidence required to guide proposed/planned actions under the Shire Valley Vector Control (Shire-Vec) research project, ultimately setting the agenda for incorporating VBD management into irrigation farming.

## 2. Methods

### 2.1. Study Design

This study employed a cross-sectional qualitative design to explore the status of vector-borne disease (VBD) control and management within irrigation schemes in Malawi. The design was chosen to capture diverse stakeholder perspectives and contextual insights into policy, implementation, and coordination challenges across sectors. Qualitative methods are particularly suited for examining complex, multi-sectoral phenomena such as VBD management in agricultural settings, where institutional dynamics, lived experiences, and policy gaps intersect [20].

The study was nested within the Shire Valley Vector Control (Shire-Vec) Project and aimed to generate evidence to inform the integration of VBD control strategies into irrigation planning and implementation. By engaging key informants from health, agriculture, research, and funding institutions, the study sought to identify opportunities, barriers, and actionable recommendations for strengthening VBD management in irrigation contexts.

An iterative, inductive approach guided data collection and analysis, allowing for the emergence of themes grounded in stakeholder narratives. This design enabled the researchers to assess the alignment (or lack thereof) between existing policies and on-the-ground practices, and to explore the feasibility of implementing Integrated Vector Management (IVM) and One Health approaches in Malawi’s irrigation schemes.

### 2.2. Study Sites and Participants

Malawi is located in southeastern Africa, along the Great East African Rift Valley region, and has a subtropical climate with two seasons, summer and winter. Summer is hot yet wet, with maximum temperatures reaching 37 degrees Celsius and annual rainfall ranging from 700 to 2500 mm. Approximately 95% of annual precipitation falls within six months (November to April), with the remainder being generally dry [21,22]. The country is divided into three regions: North, Central, and South. Malawi’s main economic activity is agriculture, which is primarily subsistence.

**Figure 1:**
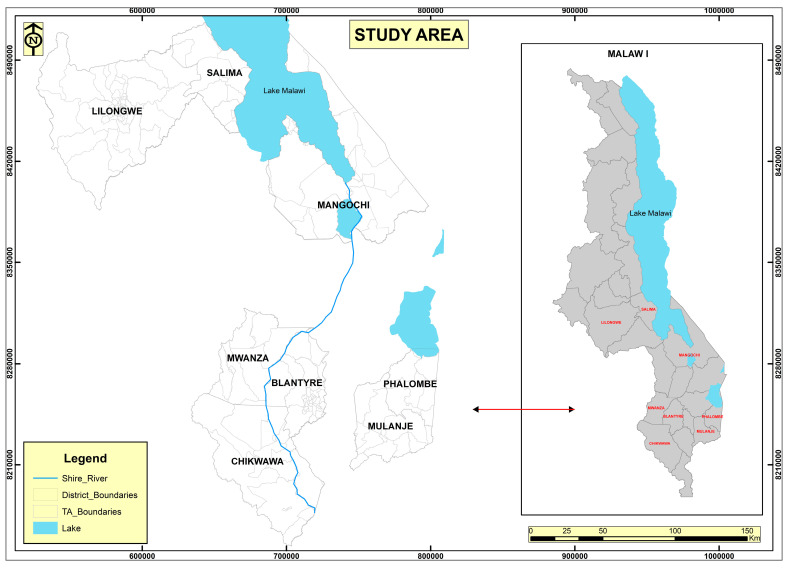
Map of Malawi showing the study area (districts).

The study was carried out in eight purposively selected districts: Lilongwe and Salima in the centre region, and Mangochi, Blantyre, Chikwawa, Phalombe, Mwanza, and Mulanje in the south. These districts were chosen based on their involvement in irrigation activities, proximity to the Shire Valley area, and participation in national vector control measures such as indoor residual spray (IRS) and mass drug administration (MDA). The study’s goal was to understand policy perceptions among institutional irrigation farming stakeholders, so participants included irrigation and VBD policymakers and implementers (those who interact directly with irrigation communities and farmers), VBD researchers, and irrigation scheme funders. Figure 1 below depicts a map of the study region.

### 2.3. Data Collection

Two experienced researchers conducted interview sessions either in-person or remotely via Zoom between October 2022 and March 2023 after obtaining written informed consent from study participants. We used a semi-structured topic guide in the English version to collect data. Prior to data collection, we piloted the interview guide with a few participants to assess its ability to collect the right information. With this process, we identified areas that required revision and incorporated the changes accordingly. The emerging data was included in the analysis. We employed an iterative process and developed new questions from the emerging gaps and explored these in subsequent interviews. Interviews were stopped when saturation was reached.

### 2.4. Data Management and Analysis

We assigned a personal identification number to each participant to maintain anonymity and excluded identifying information from the quotes. Interviews were conducted in English and recorded with permission and transcribed verbatim. Transcripts were read multiple times to facilitate data immersion and gain a deeper understanding of participants’ experiences and perspectives. To guide the analysis, a codebook was developed iteratively, and similar codes were grouped into categories to reduce and organise the data into manageable segments. These categories were then synthesised into overarching themes. We used QSR NVivo 12 software to support the analysis, applying Braun and Clarke’s thematic approach to identify patterns across narratives [20].

### 2.5. Ethics Approval

The National Health Sciences Research Committee (NHSRC) reviewed and approved the study after obtaining permission from all the participating districts. All participants were above 18 and of sound mind. To maintain the privacy of participants, we secured signed consent from all respondents before any study procedures were performed. Participation was on a voluntary basis, and interviewees were informed they could stop or interrupt the interview at any point. All audios and transcripts were saved in a password-protected computer with limited access.

## 3. Results

### 3.1. Characteristics of Study Participants

Four key VBD and irrigation stakeholder groups, comprising VBD researchers, irrigation project funders, policymakers, and district policy implementers from the Ministry of Agriculture, Irrigation and Water Development (MoAIWD), and the Ministry of Health (MoH), were identified in Malawi. We purposely selected key informants from this pool to answer questions derived from the study topic. Participants were drawn from both national level (policymakers in ministries of health and agriculture, funders and researchers) and sub-national levels, district environmental officers, malaria programme coordinators, and district irrigation engineers. A total of 21 participants were interviewed (Table 1).

### 3.2. Participants’ Views on Integrating VBDs in Irrigation Schemes

All participants affirmed the importance of tackling VBDs in irrigation schemes, given VBDs’ negative impact on farmers and their households, thereby affecting productivity. An MoH official explained how VBDs have a negative impact on both the health sector and the agricultural sector:

“*To them (MoAIWD), they intended to have this individual in the field for crop production, whether it’s growing rice or maize. But when they are sick, they are away from the field, and so there will be low production. That affects us in that we would have to procure drugs for the sick and continuously go to investigate how the person got infected*.”

### 3.3. VBD Control and Management Policies in the Irrigation Sector

Irrigation engineers from the MoAIWD described the irrigation-related policies that they were aware of. The documents mentioned included the Irrigation Policy, the Irrigation Master Plan, the Environmental Management Policy, the Water Policy, and the Agricultural Sector-Wide Approach. As climate events (floods and droughts) have become more frequent, the need to implement interventions to counter the effects of climate change has necessitated policy formulation. Irrigation has also been seen as urgent to meet the food needs of a rapidly growing population and to bring in cash income at the household and national levels. The goal of the Irrigation Policy was described as follows by two different district-level irrigation officers interviewed:

“*To contribute to the sustainable national economic growth and development through enhanced irrigated agriculture productivity. We want to increase land under sustainable irrigation farming, facilitate crop diversification, and monitor business culture in small-scale irrigated agriculture. We want to create an enabling environment for irrigated agriculture*.”

“*Increase land under sustainable irrigation farming, facilitate crop diversification and intensification, create an enabling environment for irrigated agriculture, optimise investment in irrigation development considering climate change, enhance capacity for irrigated agriculture and promote a business culture in the small-scale irrigated agriculture*.”

#### 3.3.1. Inclusion of VBD Control and Management in Irrigation Policies

Overall, according to respondents from MoAIWD, there was consensus that there was no specific provision for the control and management of VBDs in irrigation policies beyond the infrastructure development phase. Despite the lack of policies addressing VBDs, a respondent from the MoAIWD stated that the code of conduct for irrigation stipulates how irrigation infrastructure should be constructed in a way that deters vector breeding. This suggests that policy is based on siloed action without articulating shared responsibility or resource distribution. One of the irrigation engineers interviewed reported that the only provision for VBD management in the agricultural policy documents is to work with the Ministry of Health.

“*The Irrigation Policy 2016 stipulates that the Ministry of Health needs to work in conjunction with the Ministry of Agriculture, Irrigation, and Water Development to provide appropriate interventions such as the promotion of hygiene and sanitation education to prevent water-related diseases or VBDs*.”

Irrigation engineers reported that VBDs often emerged as a problem during environmental impact assessments and noted that it was possible that VBDs were considered during the planning and construction stage but were not investigated any further.

Regarding the involvement of irrigation engineers in the policymaking process, this depended mainly on how long they had been with the civil service. Some respondents stated that they were involved in the development of the National Agricultural Policy, the National Irrigation Policy, the Master Plan, and, more recently, the evaluation of the National Agricultural Policy. It was the general view that the formulation of policy in this area should be under MoAIWD, but the government must consult other stakeholders.

#### 3.3.2. Inclusion of VBD Control and Management in Health Sector Policies

Like the agricultural sector, respondents from the MoH, though they have the National Health Policy, reported lacking an all-encompassing policy or guidelines for VBD control and management. Many of the respondents cited the Malaria Strategic Plan, which makes provisions for indoor residual spraying and long-lasting mosquito nets. Program managers for specific VBDs (Schistosomiasis and Lymphatic Filariasis) reported that they used the WHO guidelines to guide them in the control of vectors, which had emerged as a problem, but there were no documents specific to the Malawian context. These guidelines make mention of VBD control, such as snail control in the case of schistosomiasis, but such interventions have not been operationalised. There was agreement on the need to harmonise vector control activities and establish a VBD department or advisory committee to inform decision-making. In the absence of a policy, MoH respondents did not feel they were the responsible party to initiate VBD control and management in the context of irrigation.

A programme manager for one of the VBDs at the MoH said, “*I think the Ministry of Agriculture needs to be engaging the Ministry of Health when it comes to issues of irrigation, for example, the green belt initiative. When they are creating irrigation canals, they are also creating breeding sites for snails, breeding sites for mosquitos, and all those vectors; so, I think they need to engage the Ministry of Health when they are doing such projects*.”

MoH respondents stated that they have not had any input into agricultural policies. However, they reported that there have been some instances when parties from the agricultural and health ministries have worked together. An official from the MoH commented on how they had been involved in the irrigation schemes in the past.

“*I remember we went to Machinga to visit some irrigation schemes. During our discussion there, we focused on the integration issues related to how best to protect our farmers from snails that can transmit diseases*.”

The MoH respondents, however, explained that the relationship with the agricultural sector is not institutionalised; there is a need to strengthen and institutionalise their linking mechanism so that they can work collaboratively in addressing VBDs. From the interviews, it appears that there have been past efforts to establish some linkages, but these have not been fruitful. A respondent from the MoH mentioned the existence of a vector control working group, which had made efforts to include the MoAIWD, but this had not been successful.

“*So, the thing is we constituted the group, which also included some experts from the agricultural section, especially from the crop husbandry within the agricultural sector. So initially they used to join us in meetings, but eventually, for whatever reasons, they just stopped*.”

### 3.4. Investments in Irrigation

Respondents from the MoAIWD reported that the government of Malawi was committed to financing irrigation, even though the bulk of the current investments is from the World Bank and the African Development Bank. The government has, however, committed to rehabilitating irrigation schemes damaged by flooding, particularly in Chikwawa. In the SVTP, the government has availed funds from phase 1 to the current second phase. They have also played an important role in providing technical advice. Further, participants noted that there is interest within the government to further expand such irrigation initiatives to other areas. Besides the World Bank and the African Development Bank, multiple non-governmental organisations (NGOs), including World Vision Malawi, Christian Aid, World Food Programme (WFP), Food and Agriculture Organisation (FAO), USAID funded projects, and local organisations such as Centre for Integrated Community Development (CICOD), Catholic Development Commission (CADECOM) and Evangelical Association of Malawi (EAM) were mentioned by irrigation engineers from MoAIWD.

#### Funding Mechanisms by the World Bank

A respondent described the World Bank funding modalities for agricultural irrigation projects in Malawi as follows:

1. The Ministry of Agriculture presents its priorities to the Ministry of Finance and allocates funding through the World Bank-funded International Development Association (IDA).
2. The World Bank develops a strategy for Malawi in which priority areas are identified that fit into the bank’s vision of shared prosperity. This is done in collaboration with the Ministry of Finance.
3. A post-disaster needs assessment is done, which looks at the impact of the disaster on the economic sectors of the country; the bank then allocates grants to sectors that have been severely affected; the projects may consist of reconstruction and rehabilitation. Significant investments have gone towards irrigation since 2015 due to flooding.
4. The last modality used is a trust fund. This entails the bank doing an analytical study for the client to help them identify areas that need financing. No investments are made.

### 3.5. Inclusion of VBD Control and Management in Budgets

Regarding whether there is provision for VBDs when financing irrigation schemes, respondents detailed that financing was provided to the government as a lump sum. The department of irrigation at the district level, therefore, determined which inputs would be used in the scheme and how the funding would be allocated. Engineers from MoAIWD indicated that VBD control and management were not initially included in the budget of irrigation schemes. An irrigation engineer said this regarding the allocation of funding towards VBD control and management:

“*We don’t apportion during the design. If there is apportionment of resources for vector-borne diseases, it is going to be done at the environmental screening stage, where we have identified that the vector-borne disease problem is going to be escalated by the system. It means this is going to be captured under the environmental and social management plan, and so it is. There is no percentage attached; it depends on the reality of the severity of this problem as we perceive it.*”

The respondent mentioned that in such cases, interventions might include the purchase and distribution of mosquito nets in areas surrounding irrigation infrastructure.

### 3.6. Challenges in Conducting VBD Control and Management Activities

The VBD activities being conducted by district environmental health offices (DEHOs) included health promotion activities on prevention and control of VBDs, such as wearing personal protective equipment (PPEs), provision of insecticide-treated nets (ITNs) (routine at health centre level and sometimes during campaigns), and mass drug administration for prevention and control of schistosomiasis and onchocerciasis. There were mixed responses regarding whether VBD control and management activities happened in different districts. It was reported that they are implemented at a small scale in Chikwawa and in Mulanje. Respondents working at MoH and the environmental health offices specifically cited the lack of resources as a key challenge they faced in conducting malaria control and management activities. For example, the distribution of mosquito nets to prevent malaria was one of the scheduled activities, but some areas may not have benefited due to inadequate numbers. When activities were conducted at the facility level, they were feasible, but fieldwork required transportation and meal allowances, and there was no provision for such activities in the budgets. Poor roads and long distances were also hindrances to activities. They reported that they relied on health surveillance assistants (HSAs) to conduct health education exercises. Additionally, a chief preventive health officer cited poor perception among the community as a factor contributing to low uptake of interventions. A health officer cited the fact that VBD control and management are not included in the irrigation policy and programmes as a major barrier.

“*So, the challenge is that in programming, that element of disease prevention in irrigation areas is not there; it is not included*.”

This respondent also reported that although awareness may be done in the community, there was no control implemented at mosquito breeding sites, and sometimes farmers did not use protective gear such as gumboots. Another challenge raised was the inadequate entomological capacity in the country. One of the medical entomologists noted that there is a gap with respect to entomological capacity in Malawi:

“*I would think about less than 20 people working as medical entomologists on the ground, and that’s really low for a country with a lot of entomology burdens and diseases that are vector-borne or parasitic-borne*.”

The entomologists expressed wanting to be involved in the policymaking process but not having the opportunities to do so. They also reflected that the approach to VBDs has been mainly curative.

### 3.7. Role of Research in VBD Control and Management

Some researchers articulated that they would like their research to ultimately improve the lives of people. The research being conducted has focused on vector resistance, and they would like their research to inform initiatives like the malaria control programme. They acknowledged that there is a long process between research and policymaking, but were aware that research had contributed to VBD control and management in the past (for example, with respect to indoor residual spray (IRS) mechanisms). Even though they provided data and evidence, the research did not always shape VBD control and management in Malawi, citing competing interests and a lack of opportunity to engage with decision-makers.

“*We advise the nation on what to do, but much of the direction they take depends on the funder*.”

An entomological researcher was asked about his role in the policymaking process, and he admitted that his role has been confined to generating data, which was often presented to other members of academia.

“*Mine has been more of a lab-based role, and then the findings are shared through dissemination conferences, and then all the interaction with the policy-makers and the researchers go through the principal investigator (PI). So, I hope the PIs are the ones that have been very much directly involved in policy-making discussions in Malawi*.”

Researchers reiterated points raised by other stakeholders that agricultural stakeholders may not be able to have a holistic view of irrigation schemes and the adverse events associated with them:

“*I think the main factor is the one that we’ve just recently talked about—to say there is a great gap in the sense that the agricultural people are just looking at food security by boosting field production through irrigation, but they are neglecting the important aspect of disease prevention. So, if we start working hand in hand, it could be better and will be beneficial to the farmers*.”

In the area of agricultural research and the contributions it has made towards policy, an irrigation officer noted a decline in agricultural research in the country, as the area is lacking support. The respondent called for an increase in funding and human resources to better position research initiatives for government decision-making in the agricultural sector.

### 3.8. Opportunities for Integration

Overall, all participants agreed that VBD control and management must be included in irrigation policies. Irrigation policies need to provide clauses on VBD control and management, and clauses that ensure that all partners, including donors, abide by guidelines on VBD control and management. This was following the observation that neither donors nor the government had initiated dialogue around VBDs. VBDs must be considered during planning, implementation, and evaluation stages. There is a need for collaboration to happen between the MoH and MoAIWD, and to be informed by research from institutions like MLW and KUHeS.

Participants from MoAIWD also emphasised the need to have baseline data to determine whether the prevalence of VBDs is increasing due to the presence of irrigation schemes. Researchers added that the evidence used should be locally generated. This would make a good case for incorporating VBD control and management in irrigation schemes.

“*But in the case of SVTP, during the feasibility studies and also the environmental impact assessment, they did a baseline survey. And I know of diseases like schistosomiasis and malaria; the issue about baseline came out strongly to determine the prevalence rate. So that when we start operating, we won’t ignore the disease incidences and not have measures to control them by saying that they were there even before the scheme came in*.”

Irrigation engineer from MoAIWD

Researchers also proposed the formation of technical working groups (TWGs) comprising members from both the health and agriculture sectors. Further, they echoed the need to train more researchers who can generate data to inform decision-making. Overall, there was a call for strong collaboration between the MoH and MoAIWD, with an emphasis on leadership from the MoH. A respondent from one of the MoH VBD programmes commented on the potential to strengthen the One Health approach, as activities that cut across sectors can be isolated. The One Health programme at the MoH is currently not fully functional due to the absence of a secretariat.

“*The aspect of One Health has been there, but what is lacking is its secretariat and resources to support it. If we have a secretariat which can be pushing issues from different sectors, that can help a lot in terms of vector control.*”

MoH participant

Respondents also noted that there are some areas where MoH and MoAIWD activities overlap, such as the use of insecticides for IRS (malaria prevention) and for pests. This might be an opportunity to potentially collaborate.

The formation of TWGs and advisory committees with experts from different backgrounds beyond the medical field, so that the approach is holistic, was also recommended. Respondents further noted the need to have a multi-sectoral approach that brings on board a wide range of stakeholders, including the private sector (as the country moves towards commercialising agriculture), NGOs (working in irrigation and on environmental issues), development partners, and research institutions to feed information into the policymaking process and knowledge translation practitioners to ensure scientific research is repackaged for laymen. They also noted the need to recruit human resources specifically for VBDs to address the current capacity gaps.

With reference to practical methods of VBD control and management, respondents proposed sensitising communities, the use of mosquito nets, the use of protective gear within schemes, and larval source management. Researchers called for the creation of an environment that is conducive to learning, so all stakeholders are aware of tools that can be used to mitigate the impact of VBDs, as well as other safety practices. This includes the involvement of research institutions in formulating policies. Further, researchers stated the importance of considering VBD issues before irrigation schemes start to operate because once they start operations, some of the decisions made are politically influenced.

The above findings are summarised in a SWOT analysis, which is presented below (Table 2).

## 4. Discussion

Three major themes emerged from the study in regard to VBD control and management in irrigation areas in Malawi. These are prioritisation of VBD control and management in irrigation areas, coordination of VBD control and management in irrigation areas, and the possibility of IVM in irrigation scheme areas.

### 4.1. Prioritisation of VBD Control and Management in Irrigation Areas

Our study found that there are no specific local VBD control and management policies in Malawi. Further, health policies relevant to agriculture omit irrigation. However, VBD control and management are mentioned in several health policies, and very few irrigation and agricultural-related policies, such as the Agricultural Sector Wide Approach Support Project II (ASWAP II) and Shire Valley Transformation Project, have an Environmental and Social Impact Assessment (ESIA). Despite having sustainable irrigation development as one of its three main priority areas, the most recent National Irrigation Policy (2024) makes no mention of VBD control and management [23]. This is particularly concerning because the lack of policy direction may lead to implementers continuing to disregard VBD control and management in irrigation areas. While the health sector is more concerned with reducing health risks, the agriculture sector, on the other hand, prioritises food security. As such, VBD control and management activities are a priority in the MoH but not the agriculture ministry, especially the irrigation department. The only time the agriculture sector takes VBD control and management into consideration is during the irrigation scheme construction phase. As a result, there are no deliberate and specific post-construction VBD control and management activities in most areas around irrigation schemes in Malawi. A similar scenario was observed at the start of a pilot integrated pest and vector management project in Sri Lanka, where it was noted that the agriculture and health sectors have different goals, and only a shared objective is what brings the goals of the two sectors together [24].

### 4.2. Coordination of VBD Control and Management in Irrigation Areas

WHO recognises the impact and risk of VBDs on the population and has developed a strategic approach (GVCR, 2017–2030) to tackle VBD control and management. This strategy is largely based on IVM concepts and stipulates that VBD control and management efforts require inter- and intra-sectoral actions and collaborations that involve actors beyond health and include agriculture, education, environment, finance, housing, tourism, transport, and water [3]. Our study discovered that, despite the available efforts on VBD control and management in Malawi, there is no clear collaboration and coordination amongst major VBD control and management stakeholders. While the Ministry of Health takes the responsibility for VBD control and management in general, there are no specific efforts directed at populations in irrigation areas despite such populations being perceived as high risk for VBDs. For example, while effective technical working groups have been seen promoting evidence use in policy and practice elsewhere, due to a lack of collaboration between the health sector and other major stakeholders, a proposal by the Partnership for Increasing the Impact of Vector Control (PIIVeC), implemented between 2018 and 2021, to establish a Technical Vector Control Advisory Group in Malawi did not materialise [25]. Further, the lack of coordination between the Ministry of Health and other sectors results in fragmented efforts and sometimes duplication of work, which results in serious inefficiencies, including resource wastage. This agrees with Golding et al., who state that running parallel vector control activities may be costly as compared to combining them, as it increases the direct costs of deploying interventions and using separate support structures for the control programmes [26].

### 4.3. Possibility of IVM in Irrigation Scheme Areas

Study participants noted the government’s commitment to invest in irrigation farming through several projects it is funding wholly or partially. They also highlighted challenges faced by VBD control and management, such as resource limitations (both material and human resources), insufficient required skill sets (entomological capacity), lack of involvement of all stakeholders, and other factors from the literature, including environmental change, insecticide resistance, and population growth [15]. Notwithstanding these, our study has shown that IVM in irrigation areas is possible at any stage of the scheme, whether planning, construction, or implementation. At the planning stage, irrigation funders indicated that, in principle, they consider issues around VBD control and management to be part of the whole during budgeting, even though no specific rate is provided towards VBD control and management since the funds are provided as a lump sum. At the construction stage, the irrigation engineers clearly stated that their designs are executed in consideration of controlling vector breeding. At the post-construction stage or irrigation farming implementation, the health personnel detailed that it is possible to specifically support communities around irrigation schemes with VBD control and management activities. The researchers highlighted the need to study more on the subject, sensitise and involve communities in the VBD control and management initiatives, and create TWGs, which would improve collaboration among all irrigation stakeholders on VBD control and management. All these are in tandem with the five key elements of IVM, which are: (1) evidence-based decision-making, (2) integrated approaches, (3) collaboration within the health sector and with other sectors, (4) advocacy, social mobilisation, and legislation, and (5) capacity-building [27]. IVM has been shown to be a highly effective approach to VBD control and management due to its complementarity [27,28,29].

While IVM has proven to be highly effective in VBD control and management, evidence shows that the VBD pathways involve the environment, humans, and animals. This illustrates a complicated dynamic of disease transmission, and neglecting any aspect may hinder effective control and management of vector-borne diseases [17]. For example, evidence indicates that human activities and climate change have compelled evolutionary changes in *Aedes aegypti* mosquito behaviour, leading to a heightened incidence of dengue disease epidemics in India [30]. With the One Health approach fostering coordination and collaboration among different sectors, bringing in a multisectoral, multilevel, and multigroup governance model makes it ideal for addressing complexities in VBD control and management [31]. Evidence from China reveals that combining IVM and the One Health approach has a considerable impact on vector population management, rural landscape transformation, wetland area expansion, and vector-borne disease incidence reduction [32]. Our study found that the One Health approach in Malawi is a familiar concept in the MoH but not in the agriculture ministry. Further, the literature review shows that One Health initiatives in Malawi are available in public health, and efforts are being made to develop a clear strategy or policy on the same [33]. However, the absence of a unit to properly operationalise the One Health ambitions is a significant impediment. Furthermore, the current One Health efforts in Malawi do not focus on VBD control and management in irrigation regions, necessitating purposeful efforts to incorporate VBD control and management into the strategy.

Two limitations need to be taken into consideration when interpreting the results of our study. First, the study did not cover all three regions of the country but only the central and southern regions. The focus was on the national policymakers from Lilongwe and district policy implementers from the districts along and around the Shire Valley. This may have left out other important would-be participants from the northern region and districts. The involvement of national-level officials from the central government in Lilongwe was intended to close that gap. Another limitation is the lack of voices from the community. Because the study focused on the policy environment, community members were not included as participants. This, however, may have influenced awareness of on-the-ground facts about VBD control and management in Malawi. Therefore, we suggest that comparable research in the future should account for this.

## 5. Conclusions and Recommendations

This study highlights critical gaps in the integration of vector-borne disease (VBD) control and management within irrigation schemes in Malawi. Despite the well-documented link between irrigation practices and increased VBD transmission—particularly malaria and schistosomiasis—there remains a lack of deliberate policy and programmatic attention to this intersection [8,10]. The expansion of irrigation, such as through the Shire Valley Transformation Project (SVTP), will create ecological conditions conducive to vector proliferation, yet VBD control is often limited to the planning and construction phases, with minimal post-construction interventions.

The absence of specific provisions for VBD control and management in the most recent National Irrigation Policy (2024) and limited cross-sectoral collaboration between the Ministry of Health (MoH) and the Ministry of Agriculture, Irrigation and Water Development (MoAIWD) reflect systemic fragmentation [23,25]. The lack of policy guidance is a major setback and therefore provides a strong basis for setting an agenda aimed at urgently addressing VBDs in irrigation schemes, as policy has been observed to guide and improve implementation [17]. While the health sector prioritises disease prevention, the agriculture sector focuses on productivity and food security, resulting in siloed approaches that undermine integrated responses. This disconnect is further exacerbated by limited entomological capacity, inadequate funding mechanisms, and the absence of technical working groups [34].

The findings underscore the potential of Integrated Vector Management (IVM) and the One Health approach as strategic frameworks for addressing VBDs in irrigation contexts. IVM promotes evidence-based, multi-sectoral collaboration and has demonstrated effectiveness in reducing disease burden when implemented holistically [27,28]. However, operationalising IVM and One Health in Malawi requires institutional commitment, policy reform, and capacity strengthening across sectors. The study also reveals that researchers and entomologists are often excluded from policy processes, limiting the translation of scientific evidence into actionable interventions—a challenge echoed in other LMICs [25,29].

Nevertheless, there is a clear commitment to irrigation farming from the government of Malawi, shown through many irrigation investments, large-scale and small-scale.

To address these challenges, we propose the following:

- **Policy Integration**: Revise irrigation and agricultural policies to explicitly include VBD control and management provisions, ensuring alignment with health sector strategies and the Global Vector Control Response (GVCR) 2017–2030 [35].
- **Institutional Collaboration**: Establish a cross-sectoral Technical Working Group (TWG) comprising representatives from MoH, MoAIWD, research institutions, NGOs, and development partners to coordinate VBD interventions in irrigation schemes. These, as observed by Berg et al., are crucial but lacking in many countries [25,34].
- **Operationalise IVM and One Health**: Implement IVM principles across all stages of irrigation projects—planning, construction, and operation—using the One Health framework to address human, animal, and environmental health interactions [17,27]. This would provide a policy framework for managing VBDs in agricultural areas.
- **Strengthen Entomological Capacity**: Invest in training and recruitment of medical entomologists and VBD researchers to support surveillance, intervention design, and policy engagement [15].
- **Evidence-Informed Decision-Making**: Promote the use of locally generated research to inform policy and practice, and ensure researchers are actively involved in policy formulation and evaluation [25,29]. The regular engagement with researchers, as well as involving them in policy formulation processes, will strengthen the use of research findings in decision-making.
- **Resource Allocation**: Ensure that irrigation investments include dedicated budgets for VBD control, particularly for preventive measures such as larval source management, protective gear, and community sensitisation [8,14].

## Figures and Tables

**Table 1:** Distribution of study participants.

Sector	Number of Participants
MoAIWD	7
MoH	10
Irrigation funders	1
Researchers	3
**Total**	**21**

**Table 2:** SWOT analysis.

Strengths	Weaknesses
Presence of policy documents and processes for policy formulation	VBD control and management not included in irrigation policies
Willingness of various sectors to collaborate	Irrigation not included in MoH policies
The availability of the environmental and social management plan (ESMP)	VBD interventions mainly curative
Commitment to make use of research findings to inform decisions about appropriate interventions	VBDs only considered at planning and construction, but not any further
Existence of Technical Working Groups in the Ministry of Health	No supervision visits by MoH
Inclusion of VBDs in health policies	Entomologists/researchers not involved in policy processes
	Preventive interventions such as distribution of ITNs, IRS not prioritised for irrigation areas
	Lack of clarity about which partners should be involved in VBD Control and management in health policies
	The role of MoAIWD in VBD control management in health policies not specified
**Opportunities**	**Threats**
Knowledge of VBD in irrigation schemes and their impact	Persistent omission of VBDs in agricultural policies, as well as omission of irrigation in the MoH policies
Availability of funding to implement the ESMP and flexibility to use the funding at the district level	Lack of collaboration between MoH and MoAIWD
Willingness to address VBDs in irrigation through the formation of appropriate structures, such as a TWG	Fragmentation of VBD control efforts
Past and current research projects that have strong advocacy for policy	
VBD control and Management are a priority in the health sector	

## Data Availability

The data used and analysed during this study are available from the corresponding author on reasonable request.

## Abbreviations

The following abbreviations and acronyms are used in this manuscript:

FISD	Foundation for Irrigation and Sustainable Development
AGCOM	Agricultural Commercialisation Project
CADECOM	Catholic Development Commission
CICOD	Centre for Integrated Community Development
DEHO	District Environmental Health Offices
EAM	Evangelical Association of Malawi
FAO	Food and Agriculture Organisation
GEF	Global Environment Facility
ITN	Insecticide-Treated Nets
IVM	Integrated Vector Management
MoAIWD	Ministry of Agriculture, Irrigation and Water Development
MoH	Ministry of Health
NHSRC	National Health Sciences Research Committee
PIIVeC	Partnership for Increasing the Impact of Vector Control
PPE	Personal Protective Equipment
Shire-Vec	Shire Valley Vector Control
SVTP	Shire Valley Transformation Project
VBD	Vector-borne diseases
WFP	World Food Programme
